# Notes on the Topography, Meteorology, and Sanitary Condition of Sinope

**Published:** 1858-07

**Authors:** J. N. Radcliffe

**Affiliations:** late of the Staff of H.H. Omar Pacha.


### ORIGINAL COMMUNICATIONS.

### NOTES ON THE TOPOGRAPHY, METEOROLOGY, AND SANITARY CONDITION OF SINOPE;

### WITH REMARKS ON THE DOMESTIC HYGIENE OF TURKEY.

By J. N. RADCLIFFE, M.R.C.S.Eng., late of the Staff of H.H. Omar Pacha.

### I. TOPOGRAPHY.

ANCIENT Sinope was best known as a stronghold and the head of an independent state; modern Sinope is best known from its position on the finest bay and safest haven on the southern coast of the Black Sea. Indeed, the Bay of Sinope is styled *"the heaven of the Black Sea"* by the Turkish and Greek sailors who frequent it; for it affords a readily accessible and safe anchorage during the heavy gales which are apt to prevail upon the coast in the winter months.

A range of precipitous mountains, wooded here and there from base to summit, and sweeping in one vast amphitheatre from south-east to west, shuts in the bay on the south and west; and a series of rolling and richly wooded hills run from the shores of the bay to the base of the mountains. On these hills, in the intervals of the dense woods, are lovely grassy slopes and red-tiled hamlets; and where, as at the bottom of the bay, the green slopes are traversed with slight fences, and the wood walls of the houses are hid amid the foliage, the scenery has a very English-like aspect. In the south, the mountains seem to approach nigh to the shore of the bay; but in the west they recede several miles inland, and their rugged summits may be seen peering, tier above tier, high in the clouds. Jutting out from the continent, and connected to it by a narrow and but slightly elevated neck of land, a bold and rocky peninsula projects into the ocean in the north-west, and, running in a direction from north-west to south-east, it shuts in the bay to the northward. Towards the ocean, this peninsula presents a series of steep and rugged cliffs, which are bare and lifeless in the winter, but in the summer they are fringed at all points with wild flowers and rich verdure. Beyond the eastern point of the peninsula, a solitary grey rock just lifts its head above the water. Towards the bay, the peninsula sweeps down to the water's edge in green slopes, which are intersected with slight ravines, down which run scanty streams; and in the easternmost ravine, at the base of the mountainous extremity of the peninsula, is ensconced a little hamlet. The sides of this ravine are covered with wood.

The town of Sinope occupies the neck of the peninsula, extending quite across it, the northern fortifications being washed by the waves of the open sea, the southern by the waters of the bay. Outside the walls, on the southern face of the peninsula, there is a large suburb, inhabited principally by Greeks.

The anchorage is opposite the town; and from the anchorage the view of the town and the peninsula is very lovely, particularly when the trees are in full foliage. The grand old walls, with their numerous towers, and the time-worn citadel, form a heavy framework, above which peer the red-tiled broad-eaved roofs and red walls of the dwelling-houses, and the white walls, blue domes, and tall minaret, of the principal mosque, the dark verdure of the bay and the myrtle tree, and the luxuriant foliage of the fig-tree. On the right, beyond the walls, the thickly scattered houses of the suburb come down to the shore; and the deep red tiles of the roofs, and the many colours of the walls, form a brilliant contrast, as they are seen among the rich foliage which grows freely in the intervals of the different buildings. Some of the houses are painted a deep red; others are of a lead colour, relieved with white; many glow with the bright yellow of the fresh unpainted wood; and many more, which have long stood the brunt of wind and weather, present the most varied hues, brown, black, and grey. This great variety of colours, mingled with the deep green of the foliage, and seen under a pure sky and a bright sun, has a singularly beautiful effect.

The peninsula, according to a rough estimate, is upwards of three miles in length, and about a mile, or somewhat more, in breadth at its broadest part. It is a huge offshoot of mountain limestone; but here and there, at the base of the north-western extremity, a ferruginous conglomerate crops out, recalling the period when Sinope was a port from which the ancient Greeks obtained a highly prized species of iron. A broad strip of marshy ground, the greater portion of which, if not the whole, is dried up during the hotter seasons of the year, runs along the whole length of the table-land on the summit. On the edges of the marshy ground, and on those portions of the table-land which are not swampy, or which are earliest dried up, as well as on the slopes of the peninsula, large crops of wheat are grown yearly; but the produce is not so great as it would be if the ground were better tilled, and the table-land on the summit carefully drained. It is related that, among other advantages of ancient Sinope as a fortified place, it was in a great measure independent of supplies from the continent during a siege, in consequence of the fruitfulness of the peninsula. This is probably no exaggeration; for, with little care, the greater portion of the surface of the peninsula might doubtless be made so productive, as to yield sufficient for the ordinary requirements of an oriental garrison and town for some time.

There are many springs of excellent water on the peninsula, and almost the whole of these springs, as is customary in the East, are carefully built in, and the water is conducted into a stone basin, while in a recess near the conduit is placed a wooden or metal drinking vessel, for the use of the wayfarer. Several of the springs give a supply of water only during the moister seasons of the year; others pour out a constant and copious stream; and from the two principal springs (one on the southern slope of the peninsula, and the other high up on the face of the north-western extremity) the town is chiefly supplied with water. Stone conduits conduct the water along the surface of the ground to the town, and distribute it to the different fountains. In consequence of imperfections in the conduits, or of the springs being insufficiently protected, the water is apt to become muddy during rainy weather, and even after a heavy shower.

The isthmus is a narrow low ridge, which slopes gently on the north and south to the water's edge. The north side of the isthmus is formed of calcareous limestone rock, which runs out some distance to sea, barely beneath the surface of the water; the south side and summit are formed of sedimentary deposit washed down from the slopes of the peninsula, or deposited by the waters of the bay. There are no remains of the harbour which, it is recorded, existed on the north side of ancient Sinope. At the present time, boats of light draught only can anchor securely in a little cove at the north-east angle of the town. A mass of limestone rock juts up near the point of junction of the isthmus with the continent, and this rock is crested by a portion of the ruins of the citadel. Several wells are sunk on the southern slope of the isthmus, within the walls of the citadel; but the water is rarely or never used except for cleansing purposes, being brackish, or otherwise offensive.

Sinope is clustered beneath its old walls. When the armed peasant from the continent visits the town, he must give up his weapons at the gates before he enters. Beyond the sandhills which cover the isthmus beneath the walls of the citadel, outside the gates, and beyond the white tombstones of the cemetery, which is almost swallowed up in the heaped up sand, but a few paces altogether, and one is, to all intents and purposes, in the country. The roads at once become narrow and uneven tracks, deep in mud, and generally impassable in rainy seasons, and rough and rugged in dry seasons.

Outside the walls, the country is most lovely. Rolling hills, richly wooded, run along towards the mountains, and in the intervals of the wood are patches of cultivated ground; while on every hill and in every valley there stand solitary houses, or a cluster of two or three, dignified with the name of a village, and a position, perhaps, on the map. The woods are thick, full of undergrowth, and wild and stunted, near the seashore; but, as the mountains are neared, dense groves of splendid trees are found, and at every step one plunges further into those forests which from time immemorial have rendered Sinope important as a port for the exportation of timber, or for shipbuilding.

In the valleys and along the seashore (particularly towards the west, in the direction of Aklimane), there are large marshes and swampy tracts of ground, the necessary accompaniments of an imperfectly cultivated but rich and well watered country; and not unfrequently, in the pallid and wasted features of the peasantry living nigh the marshes, may be traced the evil effects of a malarious atmosphere. When the wind sets in from the north-west, the malaria from the marshes on the west is blown over the hills contiguous to the town; and intermittent fever not unfrequently occurs, as a consequence, in a clump of houses which is beautifully situated on a knoll just beyond the sandhills, and overlooking the bay.

The country abounds with game. Between November and April, wild boar may be found in the woods; wild ducks and swans in the marshy grounds; bustards, large and small, upon the bare hills; snipe, quail, and other game, in the valleys and on the hill sides; and the streams and the bay teem with fish.

### II. METEOROLOGY.*

I was stationed at Sinope from the commencement of November 1855 until May 1856, and, during the principal portion of that period, I noted such meteorological changes as the very imperfect means at my command enabled me to do. The only meteorological instruments which I possessed were two Fahrenheit's thermometers (one being a very excellent one by Newman), and a marine barometer, the action of which has proved to be very creditable, on being very kindly compared by Messrs. Negretti and Zambra with their standard baro-

* Abstract of a paper read before the British Meteorological Society, March 24th, 1857.

meter, since my return to England. I registered the indications of these instruments at stated intervals daily, as well as the direction and approximative force of the wind, the amount of cloud, and general characteristics of the weather; and, although my observations apply solely to the greater fluctuations of the atmosphere and of temperature, to the ordinary changes of the weather, and to the conduct of the barometer during storms and winds, yet they may possess some interest, in the absence of any other observations from Sinope, and in so far as they refer to the almost proverbial storms of the vexed Euxine.

The house in which I was quartered, and in which the observations were made, was situated about forty feet above the level of the sea, near the centre of the Greek suburbs; and it was well sheltered from the winds. The thermometers were, from necessity, suspended about twenty feet above the level of the ground, and they were attached to the wood walls of the house exteriorly. The instruments were sheltered from the direct rays of the sun, and from the rain; but their position would necessarily expose them to radiation from the walls of the building: hence their indications would doubtless be in excess of what would have been noted under more favourable circumstances. The barometer was suspended in the apartment I commonly used, at an elevation also of about twenty feet above the level of the ground; but the necessary corrections for temperature, and also for capillarity and index error, have been made, and the observations reduced to their legitimate quantity. The bold headland formed by the western extremity of the peninsula was apt to deflect the winds from certain quarters, particularly from the eastward, and thus lead to an erroneous opinion of the direction in which the wind blew by an observer in the town; but I endeavoured to correct any errors which might arise from this source by comparing my observations with the logs of ships anchored in the bay.

1. The winter of 1855-56 was regarded by the inhabitants of Sinope as milder than usual: there was less cold, less snow, and less frost. But such a season as was then experienced was no uncommon event; and it was said that Sinope at all times escaped from the severe winters which prevail along the coast eastward of the bay.

Sheltered by the peninsula from the north-east winds, perhaps the coldest winds which blow in the south-eastern division of the Black Sea; shut in, in great measure, from southerly winds, by a range of mountains; and resting upon a narrow isthmus between two broad expanses of water,—

Sinope is rarely situated for obtaining a very equable temperature. It might be predicated, that its summers would be cooler and its winters warmer than those of contiguous towns on the coast; and the experience of the inhabitants would seem to show that such is the case.

On no day during the winter of 1855-56 did the thermometer indicate a freezing temperature throughout the hours of observation. The lowest temperatures observed between morning and night occurred on the 5th of March, when, at 9 A.M., 4 P.M., and 10 P.M. respectively, the thermometer stood at 33°, 33.3°, and 30.5° Fahr. February and March were the two severest months of the winter; and it is stated that the brunt of the cold season generally falls upon these months. With April comes fine weather; and in May spring is fully developed with the heat and almost with the verdure of summer. The latitude of Sinope is 42° 2′ 2″ North; the longitude, 35° 12′ 5″ East.

2. The following is a summary of the barometrical and thermometrical means, the prevalent winds, and the weather, during December 1855, and January, February, March, and April, 1856.

December 1855. Barometer—*mean* of sixty observations at 9 A.M. and 4 P.M., 29·443; *minimum*, observed December 14th, 29·43; *maximum*, observed December 20th, 30·40; observed range, ·97. Thermometer—*mean* of sixty observations in the shade at 9 A.M. and 4 P.M., 49° Fahr.; *minimum*, observed December 30th, 38°; *maximum*, observed December 7th, 66°; observed range, 28°. Cloud—*mean* amount of, 6·05 (0—10). Winds—N.W., 10; N.E., 4; S.E., 5; E.S.E., 1; S.S.E., 1; S., 1; S.S.W., 1; S.W., 2; variable, 1. Rain fell on thirteen days, principally in showers, more or less heavy. There was only one day in which the rain continued from morning until night. Snow fell in showers on four days; and hail fell on one day. On the night of the 17th, there were several flashes of lightning, and a few peals of thunder, the wind being squally at the time.

On the morning of the 15th of December, the summits of the mountains south of Sinope were covered with snow, and it had not entirely disappeared, still remaining on the loftier peaks and ridges, when my observations ceased on April 30th.

January 1856. Barometer—*mean* of thirty-seven observations at 9 A.M. and 4 P.M., 29·999; *minimum*, observed January 31st, 29·65; *maximum*, observed January 15th, 30·39; observed range, ·64. Thermometer—*mean* of thirty-seven observations in the shade at 9 A.M. and 4 P.M., 49°; *minimum*, observed January 15th, 33°; *maximum,* observed January 31st, 74°; observed range, 41°. Cloud—*mean* amount of, 5. Winds—N.E., 6; S.W., 4; variable, 10. Rain fell in showers on five days. Snow fell on two days, once lying two days. There was an interruption to the barometrical and thermometrical observations from the 20th to the 30th inclusive.

February 1856. Barometer—*mean* of seventy-seven observations at 9 A.M., 4 P.M., and 10 P.M., 29·934; *minimum,* observed February 1st, 29·50; *maximum,* observed February 5th, 30·38; observed range, ·88. Thermometer—*mean* of seventy-seven observations in the shade, at 9 A.M., 4 P.M., and 10 P.M., 46°; *minimum,* observed February 19th, 33°; *maximum,* observed February 12th, 64·5°; observed range, 31·5°. Cloud—mean amount of, 5·9. Winds—N.W., 8; N.E., 3; E., 1; S.E., 4; S.W., 1; variable, 13. Rain fell in showers on nine days; snow fell in showers on six days; and hail fell on one day. The first film of ice which I observed on the pools near my quarters was on the morning of the 20th.

March 1856. Barometer—*mean* of seventy observations at 9 A.M., 4 P.M., and 10 P.M., 30·038; *minimum,* observed March 30th, 29·56; *maximum,* observed March 15th, 30·40; observed range, ·84. Thermometer—*mean* of seventy observations in the shade, at 9 A.M., 4 P.M., and 10 P.M., 41·3°; *minimum,* observed March 5th, 30·5°; *maximum,* observed March 13th, 55°; observed range, 24·5°. Cloud—mean amount of, 6·9. Winds—N.W., 7; N.N.W., 2; N., 3; N.E., 1; E., 1; E.S.E., 1; S.E., 5; S., 7; variable, 12. Rain fell in showers on five days; snow fell in showers on seven days, and almost continuously on two days. During the night of the 7th, and early in the morning of the 8th, five inches of snow fell; on the 10th, a thaw commenced; and on the 11th, the snow disappeared. Sleet fell on two days, and hail fell on one day.

April 1856. Barometer—*mean* of seventy-four observations at 9 A.M., 4 P.M., and 10 P.M., 29·981; *minimum,* observed April 28th, 29·66; *maximum,* observed April 2nd, 30·21; observed range, ·55. Thermometer—*mean* of seventy-four observations in the shade, at 9 A.M., 4 P.M., and 10 P.M., 51°; *minimum,* observed April 3rd, 37°; *maximum,* observed April 17th, 60°; observed range, 23°. Cloud—mean amount of, 5·5. Winds—N.W., 13; N.E., 2; S., 9·3; S.E., 10; variable, 1. Rain fell in showers on eight days, and on one day it continued throughout the day. Snow fell on the night of the 2nd to the depth of four inches, and on the 3rd and 4th slight showers fell, but on the 5th the snow had disappeared. Hail fell on two days; and on the afternoon of the 11th there occurred lightning, with a few peals of thunder and a variable wind.

3. There was a marked general correspondence between the rise and fall of the barometer and the variations of temperature, the mercury rising as the temperature fell, and falling as it rose.

During 59 northerly winds, the barometer rose in 45, sank in 10, and was stationary in 4. During 31 southerly winds, the barometer sank in 26, and rose in 5. During 3 east winds, the barometer sank in 1, and rose in 2; and during 37 variable winds, the barometer sank in 22, and rose in 15.

The northerly winds being the *cold*, and the southerly winds the *warm* winds, and the atmosphere being heavier during the prevalence of the former than of the latter, it follows that the conduct of the barometer in regard to these winds will be similar to that observed with regard to variations of temperature. The main exceptions to the rise or fall of the barometer during the prevalence of northerly or southerly winds coincided with the formation or passage of storms.

4. Eight gales passed over Sinope during the period of observation. Of these gales, seven were preceded by a fall of the barometer, and one by a stationary barometer, during twenty-four hours. Five of the gales occurred during the night; one in the evening; one in the afternoon and evening; and one during the night and following morning. The duration of none of the gales exceeded thirteen hours. Two of the gales were from the north-east, four from the north-west, and two from the south-east; and two occurred in November, one in January, four in February, and one in March.

The conduct of the barometer and the veering of the wind preceding and following the gales would seem to indicate that they were revolving storms; and the brief duration of the storms, and their rapidity of access, lead to the supposition that they were storms of comparatively contracted area, and that they depended in the main upon atmospheric changes occurring upon the coast.*

---

* Towards the termination of December 1855, as the *Rajah Rajah Swarree*, a teak built vessel of 600 tons, was approaching the Straits of Kertch, with most of her sails set, a squall suddenly swept up from the eastern coast (coming from the "bed of Boreas", as the ancient Greeks termed the Caucasian mountains), and carried away the whole of the three masts, leaving the vessel a complete wreck. Ships under easy sail were visible from the wreck, a few miles both a-head and a-stern; but they were not affected by the squall. The *Rajah Rajah Swarree* drifted directly south until she reached the Anatolian coast, a little to the east of Samsoun, when her anchors were dropped, and she was picked up by a coasting steamer and brought into Sinope.

The principal circumstances attending each gale are set forth in the accompanying table. (p. 154.)

5. Squally weather occurred six times with a rising barometer, four times with a falling barometer, and once at the apex of a rise. The weather became squally once in November, five times in December, once in January, once in March, and thrice in April. With a rising barometer, the squalls came up from the N.W thrice, from the N.E. twice, and from the S.E. once; with a falling barometer, from the S.S.W. once, from the N.W. twice, and from the N.E. and N. once; and at the apex of a rise, from the N.E. once.

6. There were seventeen depressions of the barometer which were more or less worthy of remark, from their extent and rapidity. Of these depressions, seven, as already noted, were followed by gales; and of the remainder, the majority occurred coincidently with an elevation (in several instances sudden) of temperature and a variable wind.

7. The barometrical curve described four greater and two smaller waves. The duration of these waves, and their order of occurrence, were as follows: a small wave commencing on December 6th, and terminating on the 12th; a large wave commencing on December 15th, and terminating on January 31st (this wave was interrupted by a considerable inflection, commencing on the 6th, and terminating on the 16th of January); a second large wave commencing on February 1st, and terminating on the 23rd; a second small wave commencing on February 23rd, and terminating on March 4th; a third large wave commencing on March 4th, and terminating on the 30th; and a fourth large wave commencing on March 30th, and which had not terminated on April 30th.

8. The climate of Sinope appears to offer great advantages to the settler or invalid who may be led to the southern shores of the Black Sea, due care being always exercised to avoid the sources of atmospheric pollution which abound within the town, and the marshy districts which are plentifully scattered outside the walls.

### III. SANITARY CONDITION.

Although Sinope has a very picturesque aspect as seen from without, it is not so picturesque within. Not that the interior is altogether devoid of beauty; for there are fragments of old fortifications; gaudily decked, yet time-worn houses; quaint fountains, traversed with ancient inscriptions, and overhung with foliage; and narrow rough streets, which would be treasures to an artist, and which would prove gems on canvas,

| No. | Direction of gale. | Date. | Period of day when it occurred. | Duration. | Barometer. Amount of depression. | | | | Time occupied by depression. | | | Wind. | |
|---|---|---|---|---|---|---|---|---|---|---|---|---|---|
| | | | | | Before gale. | During gale. | After gale. | Total amount. | Before gale. | During gale. | After gale. | Before. | After. |
| | | | | hours. | | | | | hours. | hours. | hours. | | |
| 1 | NE. | 27 Nov. | Evening. | 2½ | ·35 | ·05 | ·11 | ·51 | 11 | 2½ | 10½ | 0 | 0 |
| 2 | NW. | 30 Nov. | Night. | 5 ? | ·17 | ·21 | ·0 | ·38 | 30 | 5 ? | 3 | NE. 4* | 0 |
| 3 | NE. | 13 Jan. | Night. | 7 | ·0 | ·0 | ·0 | ·0 | .. | .. | .. | SW. 6 | NE. 5 |
| 4 | NW. | 9 Feb. | Afternoon and evening. | 12 | ·33 | ·0 | ·0 | ·33 | 55 | .. | .. | E. and NNE. 1 | 0 and SE. 1 |
| 5 | NW. | 12 Feb. | Night and morning. | 12 | ·36 | ·0 | ·0 | ·36 | 35 | .. | .. | SE. 1 | NW. 1-5, and 0 |
| 6 | SE. | 20 Feb. | Evening and night. | 12 | ·14 | ·13 | ·0 | ·27 | 18 | 6 | .. | NE. 2 | E. to NE. and SE. 1 |
| 7 | SE. | 22 Feb. | Night. | 8 | ·33 | ·13 | ·0 | ·46 | 24 | 8 | .. | E. to NE. and SE. 1 | E. to NE. 1 |
| 8 | NW. | 4 Mar. | Night. | 13 | ·22 | ·0 | ·0 | ·22 | 31 | .. | .. | SSW. W. to N. 1 | SW. and S. 3 |

* Force, 0—10.

where mud forms a capital foreground, and tumble-down buildings look picturesque in their ruin, but not wretched; and foul odours are altogether wanting.

The houses are all built upon the same plan. This is, a basement story formed of a strong framework of wood, the interspaces being built up with stone, generally undressed; and over the basement is erected a story built of wood, either in good three-fourths-of-an-inch plank, as in the better class of houses, or of broad laths, as in the inferior class. Some of the best houses are built throughout of brick or stone, in the interspaces of the wood framework. Most of the houses are painted externally; all have broad eaves; and in nearly all the second story projects beyond the basement, and overhangs the street, approximating tolerably closely to the jutting portions of the houses on the opposite side. In some houses, that part of the second story which projects above the street is converted into a species of balcony. The windows of the better houses are glazed, and, in the houses of Mussulmen, latticed; but, in the commoner houses, the windows are unglazed, and simply closed by shutters.

The projection of the upper stories of the houses above the street is an important adjunct to the picturesque aspect of the town, but it has one great drawback. It is very customary both with Turks and Greeks to cast anything which may have to be rejected through the window into the street. Occasionally, also, the spouts leading from the sinks of the houses project high above the head, towards the centre of the roadway. Moreover, the floors of many of the projecting stories are formed of planks fixed so widely apart that it is simply necessary to cast water upon them to get easily rid of it; and when they are cleansed, a shower of a most unpleasant description usually falls into the street. Hence it happens that a sharp look-out is required in passing along the streets, in order to avoid being deluged with filthy fluids cast from above.

Many of the houses, particularly those newly built and painted, have an exceedingly neat and clean appearance, and their construction is not without taste. The eaves are ornamented with mouldings; the supporting frames and window frames are slightly decorated; and the whole of these ornamented portions are painted white. In the higher class of houses, the under portions of the eaves are decorated in a somewhat pretentious fashion; and, in some of the older houses, it is not uncommon to see beneath the eaves an elaborately designed and very elegant depending cornice, which at one time had been gaily painted in divers colours. But it is difficult to conceive the wretched, ruinous aspect of the older houses which are denuded of paint, and of which the wood is mossy-grey or black with age, and is traversed with broad gaps and rents. This is indeed the condition of the majority of Turkish houses; and it is the predominance of such dwellings which is the chief characteristic of a Turkish town, and which gives to it its peculiar tumble-down and miserable aspect when viewed close at hand.

There is a singularity about the houses of all classes in Sinope, in the addition of small windows, variously, and often elegantly designed, and filled with glass, which is not unfrequently fancifully coloured. These windows are fixed above the ordinary windows of the house, and it is not uncommon to see several in one apartment.

The roofs of the houses, which are invariably tiled, have also stuck upon them, commonly in the most conspicuous part, an ornamented and variously designed appendix.

The streets are narrow, paved irregularly with boulders and stones, interrupted plentifully with deep holes, and in the centre runs the main sewer, towards which small rills of fœtid, offensive matter flow sluggishly from the houses on each side.

The houses are entered, generally, by large folding doors, which open into the basement story. This story is used indifferently as a stable, a cow-house, or a store-house, and not unfrequently within it, or communicating with it, or at the most placed just without, nigh to a door leading from it, is an enormous cesspool, connected with the privy. A few planks, or a stone slab or two, having an aperture of communication, being placed above the cesspool; or a connexion being made with it by means of earthen tubes, conducted to the upper floors of a building, as in first-class houses, forms all the fitting up of a Turkish privy. Ascending from the basement story by a flight of wood steps the second story is reached, which is occupied by the family. In comparatively new houses, where the wood is not discoloured by time, the appearance of this story is invariably most pleasing. The walls are usually arranged in neat, oblong panels, the rooms have a plentiful supply of cupboards and other receptacles about them, and frequently a narrow platform, or divan, partially surrounds the apartments, or is placed in a recess, beneath a wood canopy, more or less ornamented. The ceiling is also of wood, panelled like the walls of the room. The bright yellow of new wood gives a very agreeable aspect to apartments thus constructed. In the higher class houses the walls are painted, and there are more vigorous attempts at decoration; festoons of singularly designed flowers being painted along the upper portions of the walls, the panels having centre pieces representing miracles of floriculture, in which roses, pinks, and tulips grow from one stem, the leaves of which are in themselves, as should be the case, exceedingly peculiar. The rooms invariably abound in windows.

In the older houses and those of the poverty-stricken, when the wood-work is time-worn and fissured, and the floor slight and badly made, the structures are little better than huge sieves, which the wind and the rain permeate freely; and the wood being blackened by time, smoke and filth, the interiors have a most wretched aspect.

Every house possesses a plot of ground in the rear. Some of these plots may perchance be cultivated in a certain fashion, but in general they are filled with rank grass, weeds, and trees, the fig, with its luxuriant foliage and wide-spreading branches, being predominant. The abundance of foliage, and its contiguity to the houses, adds greatly to the picturesque aspect of a Turkish town, and relieves somewhat the wretched flimsiness which else would be so apparent in a series of wood houses crowded together.

Doubtless, as I can testify from personal experience, a wood house is, when newly built, a very satisfactory and pleasing residence, but, sooner or later, the watchful eye will detect sundry minute spots in the neighbourhood of the crevices of the wood in the different rooms, and then one may bid farewell to all persistent comfort, unless he have, by good hap, obtuse feelings, or be a native; for at nightfall there will be seen to issue from the crannies legions of bugs of gigantic stature and of ferocious character. The houses are, indeed, infested with these abominable insects and with fleas, and that to a degree which is almost inconceivable, but a native never seems to be in the least inconvenienced by them. They take no steps to mitigate the plague, or rather to prevent the development of these insects, for a plague they are not to the natives, as they seem to suffer very little from them, and one is almost involuntarily led to the conclusion that they would be uncomfortable without them.

Bugs and fleas seem to be nuisances almost inseparable from buildings constructed of wood, after the fashion of Oriental houses, and their presence is not necessarily indicative of filthy habits on the part of the people.

Turkish houses and, in consequence, Turkish towns contain within them, in a concentrated form, the majority of those things which tend to make houses and towns unhealthy. The invariable cesspool, which is within or contiguous to the basement of each house, constantly emits a stream of effluvium which, in certain states of the atmosphere, becomes intolerably offensive.* In addition to this, the offscourings of the house are cast into the street, or they are thrown upon the surface of the ground in the open space adjoining the house, where they accumulate, or the surplus runs off along the sides of the building until it reaches the common sewer, the street, and from the surface of the ground thus saturated with decomposing matter, and from the confined streets, noxious emanations are being continually given off. That the health of the persons exposed to an atmosphere polluted from such sources, particularly when in a concentrated state, as in Turkish towns, will be more or less deteriorated seems highly probable; nevertheless, in the main, there is no very palpable evidence (except, perhaps, in the most densely inhabited portions of the larger towns and cities) of such deterioration. This may be accounted for by the considerations, that the families always live in the upper stories of the houses, at some elevation above the ground; that there is commonly an open space adjoining the house, admitting of free ventilation by the access of the winds; and that the habits of the people, both in diet and labour, are temperate.

Sinope is notorious on the coast for its healthiness, not only as compared with other towns on the southern shore of the Black Sea, but also on account of its (reputed) singular exemption from epidemic diseases. No doubt there is a great deal of truth in this notoriety, for the elevation of the town is such as to ensure to it an exceedingly delightful climate. It is at all times a most difficult task to obtain any statistical returns of death and sickness from the Turkish authorities, and little faith is to be placed in such returns as may be got unless there may be some collateral method of testing them. I obtained from the Registrar of Sinope the following returns of the male population above six months of age (the Turkish census being, I believe, solely confined to that portion of the population) and of the deaths among them.

| | |
|---|---|
| Male Turks (from six months of age and upwards) within the walls | 1185 |
| Ditto, ditto, outside the walls - - - - - | 267 |
| Male Greeks (from six months of age and upwards) last return 988, but now increased in number, say - - - - | 1030 |
| Total - - - - - - | 2482 |

* The effluvium from the cesspool is not the only nuisance to which it gives rise; for the cesspool of the house in which I was quartered formed a huge nidus for the development of *millipedes*, and, during the spring, the walls of the basement story of the building, within, and of the room which I occupied, were infested by these offensive looking myriapoda in a most disgusting fashion.

The *deaths* among the *male* population above six months of age average from 20 to 30 every year, and rarely exceed 40. This gives a proportion of rather more than *one* death in every *eighty-three* individuals of the male population, at the ages returned, annually. This is a very small mortality, if it be correct, but of this I doubt, from the extremely careless manner in which the records are kept. Moreover, from these returns we obtain no notion of the general rate of mortality among the whole population.

The registrar asserted that during the two months January and February, 1856, the mortality among the male population had been excessive, and he considered that the number of deaths equalled that of the total mortality of the three or four preceding years. It was during the two months named that I had an opportunity more particularly of witnessing the forms and progress of disease in Sinope, and I was forcibly struck with their resemblance to the low types of disease in our large towns. But Sinope had for many months been subject to conditions which were unusual to it. The principal dépôt of the British land transport corps and a commissariat dépôt for cattle were established in the town, and it was inundated with men attached to the dépôts. The resources of the town and country were insufficient to meet the requirements of this great influx of persons (amounting to about 3,500, the principal portion of whom were quartered in huts, together with about 7,000 animals—horses, mules, &c., just on the verge of the suburbs, to the north), and during the winter there was a scarcity of provisions among the inhabitants of the town, and though they were accumulating money to an extent which might almost have satisfied even Greek cravings after pelf, they were unable to obtain any regular supply of meat and vegetables.

The sanitary deficiencies of the town were also much exaggerated while it was thus overcrowded, particularly during the summer and autumn of 1855, when a murrain prevailed among the cattle in the commissariat dépôt, and in the town and country adjoining; and whilst it continued, putrefying carcases of animals which had succumbed to the disease were scattered thickly about the town and suburbs, and the air was filled with foul odours.*

* The murrain, which broke out at Sinope in the summer of 1855, prevailed widely in Asia Minor during the same year. The disease was characterised by a profuse and suddenly set up diarrhœa, and an animal rarely survived more than six hours after it was attacked. The disease resembled cholera in many particulars. For an account of the murrain as it occurred at Sinope, see a paper which I communicated to the Epidemiological Society,

### IV. HABITS OF THE PEOPLE.

I was quartered in the house of a Greek storekeeper at Sinope. My landlord's household consisted of himself, his wife, a dubious elderly personage (who was either a sister, or aunt, or a mother of one of the heads of the house), a sister-in-law, and three children. Now, although the house contained four rooms, the whole of the family were accustomed to live and sleep in one of the rooms only. This crowding together of families in one apartment seems to be common among the lower classes of the East. I found it prevail to a great degree in Sinope and its vicinity, and to have no relation whatever to want of space; for it was as usual among those families who occupied large houses as those who occupied small ones. The inconveniences which might seem to arise from so many persons occupying one apartment, are certainly diminished to their least degree by the mode of life and the simplicity of the household furniture. Their cushions and carpets, which serve for seats by day and for beds at night, are the only furniture of an apartment: hence, the whole area of a room is at complete disposal, there being no tables or chairs to take up space unnecessarily. At meals all eat from the same dish, and each person being provided with a spoon and a piece of bread only, very little room is required to accommodate a tolerably large party. A circular stand, about six inches in height, is placed in the centre of the floor, the requisite number of spoons and pieces of bread are laid upon it, and the dish containing food stands in the centre. Squatted at this low table, each in succession dips his spoon in the dish, and less than half the lateral space is required for eating comfortably than when a knife and fork are used. Even when a family is large, and the crowding greatest, and when persons of all ages, married and unmarried, sleep in the same room, it seems to lead to little or none of that impropriety of thought or immorality which is witnessed, under similar circumstances, in our own country. That ill-directed energy of thought which results, particularly in the ignorant, from much association, and from that extreme freedom of intercourse which prevails among the young with us, is altogether wanting in Asia Minor; and beyond the great towns, and, indeed, in them there would appear to be little immorality among the young of different sexes.

The crowding together of families has, moreover, certain

---

and which is printed in the Appendix to Dr. Greenhow's "Report on Murrain in Horned Cattle", *Blue Book*, 1857.

advantages; for in the colder seasons of the year, it tends materially to maintain the warmth necessary for comfort during the inaction of the night. The oriental has a most imperfect notion of the use of fire for any other purpose than that of cooking victuals. The manner in which he manages a fire for cooking is a perfect study. With a handful of embers and a few sticks he will keep his fire in a constant glow, and contrive to cook several dishes at the same time in the most delightful fashion. The fact is, that he cooks the majority of his dishes with a heat short of the boiling point—the great secret of cooking most things well. Thus it happens that he uses little fire, is an invaluable servant during a campaign, and is readily taught to become a capital cook; but he comprehends only in a slight degree the use of fire for warmth. It matters not that he may have a superabundance of wood: I have seen Greeks and Turks, in bitterly cold nights, wrapped up in their great coats, and shivering over a handful of half-extinguished embers, when an unlimited supply of wood has been within a few feet of them. Ignited charcoal is the favourite agent with the oriental for heating rooms during the winter. The charcoal is fully ignited in the open air upon a stand, made of earthenware or brass, and called a *mangal;* when the charcoal has attained a white heat (for previous to this it gives off dangerous fumes) it is carried into the apartment. One or two of these *mangals,* bearing incandescent charcoal, are used according to the size of the room, and the ignited charcoal is replenished as often as may be necessary. The family sit round the *mangal,* eking out the heat which it gives off by additional clothing, the men burying themselves beneath large overcoats, lined with sheep-skin or fur. During very cold weather, when one has nothing to do but to crouch over the *mangal,* enveloped in any amount of clothing, and to hunt after the heat, doubtless it may be found to a certain extent; and again, with a sufficiency of *mangals* a room may be actually warmed; but under ordinary circumstances the *mangal* is a grave delusion, and to an Englishman, who delights to throw all upper clothing aside when he enters an apartment, it is a bitter snare. The heat which a *mangal* gives off is generally accompanied with a faint and unpleasant smell, and it is also of so oppressive a character that it takes some time to get accustomed to, and to tolerate it with comfort. But a good fire blazing upon the hearth and beneath the large chimney (if the room possess a hearth and chimney), or in a capacious stove, is not a sure defence against cold in a Turkish house, as there are so many ways by which the external air can get entrance into the flimsy wood buildings that at times the cold will set the warmth at nought.

The long coat of sheep-skin, or thick cloth lined with fur, which is commonly worn by all classes of men in Turkey, is an admirable article of dress, and it is an excellent plan for the traveller to adopt it, and to follow the oriental custom of wearing the coat in the early morning, during the evening, and when rain is falling, in the finer seasons, when inactive. For under the hot sun of Turkey the skin acts excessively, and becomes very susceptible of morbific influences; and when towards evening lassitude and languor steal upon one, and the cool evening breeze is revelled in; or after a hot night, when the morning breeze is gladly welcomed, the reduced temperature caused by the wind, slight though it may be, is sufficient to act unfavourably upon the over-excited skin, and to induce diseases of a very formidable kind. The adoption of the long-coat, which is too loose to incommode one when at rest, and which may be wrapped round one so as to shield without occasioning undue warmth or inconvenience, at once diminishes the chance of harm from this source very greatly. Englishmen are exceedingly apt to suffer from carelessness in this respect. It is particularly delightful in an evening during summer to lounge before the open window, or upon the soft sward, with the dress thrown open, or in part cast aside, and to experience the delicious sensations excited by the breeze after an intensely hot day. But how common it is for an exposure of this kind to be followed by rheumatism, fever, or diarrhœa! It is never to be forgotten that the wind, in addition to the evils which it may occasion by a diminution of temperature acting upon a susceptible surface, in most parts of the East sweeps over tracts of uncultivated, or imperfectly cultivated, land, or marshes, and it is too apt to bear insidious poison upon its wings, and to find the traveller, particularly if he has been abiding for any lengthened period in the vitiated atmosphere of an Eastern town, an easy prey. Nay, even the sea-breeze is not to be trusted heedlessly, for some of the foulest and most malarious portions of Asia Minor extend in narrow strips along the sea-coast.

Most Turks and Greeks sheathe the skin from the neck to the knees or ankles with cotton, and it is a very necessary precaution in the East to wear under-garments of cotton or flannel, as a protection against sudden diminutions of temperature (positive or relative), particularly during the hotter seasons of the year when the inordinately excited skin is highly sensitive to changes of temperature. There is another custom, which is perhaps most common among the Turks, and which may also be wisely followed, and that is the spreading of a carpet between one and the ground when sitting in the open air upon the sward.

Cleanliness is a marked characteristic of a Turkish or Greek house within, notwithstanding the profusion of insects, and notwithstanding the filth without. The latter is suffered from ignorance of its effects; the former is an evil, perhaps, inseparable from wood buildings. It does not do to compare the notions of cleanliness of an English with those of a Greek or Turkish housewife. The former seeks the extreme of cleanliness through a multiplicity of dishcloths and towels; the latter simplifies the matter somewhat, and probably sees no reason why one cloth should not do all the duty of cleansing the whole of the vessels in the establishment. Greeks and Turks are, however, essentially cleanly in their houses and persons, under ordinary circumstances. Indeed, personal cleanliness is a supreme law among Mussulmen, and the modern Greek appears to have a high notion of its virtue. Tradition records that Mahomed asserted that *the practice of religion was founded on cleanliness, and that it is the one-half of faith, and the key of prayer.*—(Sale). Among the poverty-stricken and those of degraded habits, a want of cleanliness may be met with, as is generally the case elsewhere; and rarely is filthiness, either of person or habitation, more repulsive than when it is seen in the East.

The value of water is, as might be expected from the precepts of the Mahomedan religion, nowhere more thoroughly appreciated than in Turkey. Fountains abound in the towns, and every care is taken to keep the aqueducts leading to them in good order; and in the country almost every spring is protected, and the water conducted into a basin. Sinope is plentifully supplied with fountains, and to each fountain is attached, by a chain, a metal drinking vessel, or a wood cup is placed in a recess of the masonry. Notwithstanding, however, the numerous fountains in Sinope, it must not be supposed that the supply of water is superabundant. It meets the uses which the inhabitants make of it; but the slender streams which run from most of the fountains, and the labour of carrying the water, prevent that free and copious use of the fluid which would be requisite for cleansing thoroughly the vicinity of the houses as well as the streets.

The method of cooking usually practised both by Turks and Greeks, and the times of eating, are certainly very excellent. But there can be little doubt that flesh-meat and vegetables enter sparingly into the diet of the bulk of the population of Turkey; and that rice, butter or sheep's-tail fat, and bread, are the staple articles of food. Rice and butter are very admirable things in their way, and a number of capital dishes may be formed from them, with the help of a few condiments, —dishes that will thoroughly satisfy the appetite, but it is doubtful whether they are sufficient to maintain a perfect state of the health, when they are made, along with bread, almost the exclusive articles of diet, flesh-meat and vegetables being used only at rare intervals. I suspected often that many cases of severe scrofulous disease which I saw in the East, and even the indolent habits and inaptitude for continued exertions of the majority of the people, depended a good deal upon the diet being mainly farinaceous. These are questions, however, which could only be dealt with satisfactorily under more favourable opportunities for observation than I possessed, and by a more systematic inquiry than I was able to make.

Stewing is the typical mode of cooking in Turkey, and although meat may not retain so much of its nutritive property when stewed as when roasted, still when joints, as a rule, are impracticable, and when it is requisite to economise to the full the meat at disposal, no form of cooking equals that of the stew-pot. The combinations which may be made in that vessel, by which meat and vegetables or farinaceous matters are brought into most pleasant and desirable connexion; the mode in which, by means of it, the proportion of meat may be kept in due and proper relation to other articles of food; and the satisfactory manner in which dishes concocted in it appeal to the palate and the stomach, are advantages which cannot be attained so well by any other method of cooking, and which go far to render of no effect, if they do not set aside altogether, the evils which might arise from a deficiency in the chemical constituents of meat subjected to such a process of preparation. It would be well if the stew-pot were more commonly used for the preparation of food in this country, particularly among the labouring classes.

The periods at which the principal meals of the day are taken are early morning and sunset. In the East, as elsewhere, the number of meals taken daily is governed by the duties of the individual. It rarely, however, exceeds three, and usually the number is confined to two; but, under any circumstances, the principal meals are eaten early in the morning and in the evening. The times of eating must necessarily be regulated by the duties of the day, but the plan which is followed by all classes in Turkey appears to satisfy well the physiological requirements of the system.

